# PP92 Enhancing Patient Engagement In Health Technology Assessment: Identification Of Barriers, Facilitators, And Proposals For Improvement

**DOI:** 10.1017/S0266462324002587

**Published:** 2025-01-07

**Authors:** Yolanda Triñanes, Patricia Gómez-Salgado, Máximo Molina-Linde, Paula Cantero-Muñoz, Carmen Martín-Gómez, Anabel Granja-Domínguez, Ana Toledo-Chávarri

## Abstract

**Introduction:**

Patient engagement in the health technology assessment (HTA) field is crucial but faces significant challenges. This study focuses on identifying the barriers and facilitators surrounding the incorporation of patients in HTA while proposing solutions from the perspective of the Patient Interest Group (GIP) within the Spanish Network of Agencies for Health Technology Assessment and Services of the National Health System (RedETS).

**Methods:**

An ad hoc questionnaire with four open-ended questions about barriers, facilitators, and proposals was developed and distributed among the GIP members through an email survey methodology. The obtained results were discussed in an open in-person meeting, and a thematic analysis of the data was conducted.

**Results:**

Fifteen people answered the questionnaire (at least one HTA researcher from each agency), and forty people participated in the face-to-face discussion. Main barriers included the perception of a lack of institutional support, insufficient participative culture, lack of time and experience, and difficulties in planning. Facilitators included activities organized within the GIP and methodological procedures and documents published. The main proposals for improvement were focused on the training and capacity building of HTA researchers and patients, institutional commitment to patient involvement, promotion of forums and activities with patient entities, and improvement of communication channels with patients and dissemination.

**Conclusions:**

Despite current challenges in patient involvement, this study highlights facilitators and proposals for improvement. Specialized training and education, along with the promotion of a participative culture, emerge as relevant strategies.

